# Effects of Electroacupuncture Combined With Psychological Intervention on Depressive Status and Contingent Negative Variation in Patients With Internet Addiction Disorder: A Randomized Controlled Trial

**DOI:** 10.3389/fpsyt.2021.722422

**Published:** 2021-11-15

**Authors:** Wei Peng, Yang Wang, Qinghong Hao, Jun Wang, Yalin Chen, Mimi Qiu, Yang Tu, Hui Li, Tianmin Zhu

**Affiliations:** ^1^School of Acupuncture and Tuina, Chengdu University of Traditional Chinese Medicine, Chengdu, China; ^2^College of Traditional Chinese Medicine, Chongqing Medical University, Chongqing, China; ^3^School of Rehabilitation and Health, Chengdu University of Traditional Chinese Medicine, Chengdu, China; ^4^School of Preclinical Medicine, Chengdu University, Chengdu, China

**Keywords:** internet addiction disorder, acupuncture, contingent negative variation, psychological intervention, cognitive-behavioral therapy, depression

## Abstract

**Background:** Depressive symptoms often accompany people with Internet addiction syndrome (IAD). Acupuncture has been found to have significant advantages in improving the severity and depressive symptoms of IAD. Contingent negative variation (CNV) is a common method to explore the mechanism of neurophysiology.

**Objective:** The purpose of this study was to observe the efficacy of electroacupuncture (EA), psychological intervention (PI), and comprehensive intervention (CI) in the treatment of depression in Internet addiction disorder (IAD), and to observe the changes of contingent negative variation (CNV) in each group.

**Methods:** One hundred and twenty subjects diagnosed with IAD were randomly assigned to the EA group, the PI group, or the CI group. They received EA, PI, or a combination of EA and PI for 40 days. The Internet Addiction Test (IAT), the Zung Self-rating Depression Scale (SDS), and the Hamilton Depression Scale (HAMD) were evaluated for all subjects at baseline, 20th, and 40th days of treatment, while CNV data were collected at baseline and 40th days of treatment.

**Results:** Three treatments effectively reduced IAT, SDS, and HAMD scores, and the intergroup comparison showed that CI was superior to EA, while EA was superior to PI. CNV results indicated that the CNV amplitude increased in all three groups of IAD patients after treatment. The CNV latency of point A and A-S2' wave area of the EA group and the CI group did not change significantly after treatment. Only the A-S2' wave area of the PI group increased significantly compared with the baseline period. In addition, IAD's IAT score was positively correlated with SDS and HAMD score at baseline but negatively correlated with CNV latency. After treatment, only the change of HAMD score in the CI group was negatively correlated with amplitude.

**Conclusion:** Our results demonstrate the efficacy of acupuncture and psychological intervention in the treatment of IAD from an electrophysiological perspective. Simultaneously, the increase in CNV amplitude might be the underlying neurophysiological mechanism by which CI improves depression and cognitive function in IAD patients.

**Clinical Trial Registration:** ClinicalTrials.gov, identifier NCT02362698.

## Introduction

Internet addiction disorder (IAD) has become a significant social problem, especially among teenagers and young adults. IAD is an impulse-control disorder of Internet behavior in the absence of addictive substances (1). Its typical symptoms are involved, including tolerance, withdrawal symptoms, large amounts of time spent online, interruption of social relations, and disorder of the biological clock (2–5). During the past decade, the global average incidence of IAD is about 6%, with the highest incidence in the Middle East (10.9%) and the lowest in Northern and Western Europe (2.6%) (6). Computers and mobile phones are the most critical learning and working tools for young people, but young people lack control over Internet use and are more likely to suffer from IAD than people of higher age (7). According to *The 44th China Statistical Report on Internet Development* released by China Internet Network Information Center (CNNIC) in 2019, by June 2019, the number of Chinese netizens had reached 854 million, an increase of 25.98 million compared with 2018. Higher Internet penetration means more people are at risk for IAD, especially students at school. According to statistics, the incidence of IAD in Chinese college students ranges from 10.1 to 12.5% (7). Therefore, it is of great significance to find a more reasonable scheme to intervene and prevent IAD.

IAD is associated with a variety of online activities, the most common of which is online gaming (8), as well as online shopping (9), online gambling (10), viewing pornography (11), et al. Internet game addiction (IGD), as the most studied type of IAD, has been included by the fifth version of the *Diagnostic and Statistical Manual of Mental Disorders* (DSM-5) (12). Cognitive-behavioral therapy (CBT), as a psychological intervention, is effective in many randomized controlled studies on IAD (13, 14). In addition to CBT, acupuncture therapy from China also showed good therapeutic effects in IAD studies (15). Previous studies have shown that IAD patients are often associated with mood disorders (16, 17). Other studies have shown that depression scale scores increase with the severity of Internet addiction, and depression has been identified as a risk factor for IAD (18, 19). Hence improving depression status is an essential aspect of IAD treatment. Many studies have shown that acupuncture can improve depression symptoms in patients (20, 21). In recent years, acupuncture has been used by Chinese clinicians in the adjuvant treatment of IAD and has made some progress (22). In our previous study, we found that acupuncture combined with CBT could effectively regulate the psychological state and improve the level of mental health of IAD patients (23). However, we had not previously focused on the effect of acupuncture on depression regulation in IAD patients. Whether the improvement of Internet addiction by acupuncture is related to the improvement of depressive symptoms remains further verified.

Contingent negative variation (CNV) is an important component of event-related potential (ERP), which is closely related to people's psychological activities such as motor preparation, expectation, attention, and motivation (24, 25). CNV is a slow negative brain wave that usually occurs in the time between a warning signal (S1) and a command signal (S2) (26). It contains two main components related to brain function: orientation wave (O-wave) and expectation wave (E-wave). O-wave (early component) is related to the process of orientation or warning, while the E-wave (late component) is related to the motion preparation (27). It is a good indicator of complex psychological activities. At present, the detection of CNV components is mainly applied in psychology, and there are also encouraging findings for some non-psychiatric diseases, such as migraine (28), Parkinson's disease (29), et al. So far, there are few CNV research on IAD. Our previous CNV research found that there were no significant differences in latency of point A, amplitude of point B, area of A-S2' wave, and occurrence rate of post-imperative negative variation (PINV) in IAD participants compared with normal people (30). Point A is usually defined as the point where the CNV wave leaves the baseline, point B is the peak point of the negative deflection of the E-wave, point C is the point where CNV returns to the baseline after S2, and the area of A-S2' wave is the area enclosed by the E-wave and the baseline (31). Another team found different results in IGD studies, showing an increase in the latency of point A in IGD patients and a significant decrease in both the mean amplitude of CNV and PINV compared with healthy controls (32). In addition, it is not clear whether acupuncture or CBT can affect the CNV index of IAD. As an electrophysiological technique that can reflect brain functional activities, the detection of CNV components can provide a new perspective for the study of therapeutic mechanism of IGD. Our CNV study was a randomized, controlled, single-blind, three-arm trial. We randomly divided the recruited IAD volunteers into electroacupuncture (EA) group, psychological intervention (PI) group, and comprehensive intervention (CI) group (EA combined with PI), and observed the efficacy of the three treatment regimens on IAD and the change of CNV. This study aimed to assess (1) whether CI was superior to EA or PI; (2) the difference between EA and PI; (3) whether EA can improve the depressive state of IAD patients; and (4) whether there was an association between changes in CNV components in IAD patients and clinical indicators.

## Materials and Methods

### Participants

Subject recruitment was conducted in affiliated Hospital of Chengdu University of Traditional Chinese Medicine, Substance dependence Clinic of Western Hospital, General Hospital of Chengdu Military Region, Southwestern University of Finance and Economics, and Chengdu University of Traditional Chinese Medicine from 2010 to 2012. This trial was conducted in accordance with the Declaration of Helsinki and approved by the Sichuan Provincial Ethics Committee for Traditional Chinese Medicine. The study protocol is registered at ClinicalTrials.gov (Identifier: NCT02362698). All subjects provided written informed consent prior to enrollment.

We referred to the self-rating Scale score of Internet addiction obtained in the previous study (31), and calculated the sample number of each group as about 35 cases through the software PASS 11 (NCSS, LLC. Kaysville, Utah, USA). Taking into account the 15% shedding rate, 40 cases per group was appropriate. Finally, 120 subjects who met the inclusion and exclusion criteria were recruited for the study. The inclusion criteria were: (1) diagnosed with IAD according to the diagnosis which accepted as a standard by the American Psychological Association in 1997 (33); (2) between 18 and 40 years of age; (3) no history of medication or psychotherapy; (4) right-handed; (5) Normal hearing and vision; and (6) voluntarily signed informed consent. Exclusion criteria were as follows: (1) a history of severe mental illness; (2) a history of drug addiction; (3) disease not suitable for acupuncture treatment, such as severe cardiovascular disease, blood disease, or tumor; (4) allergy to acupuncture treatment; and (5) pregnant, nursing, or lactating.

**Figure 1:**
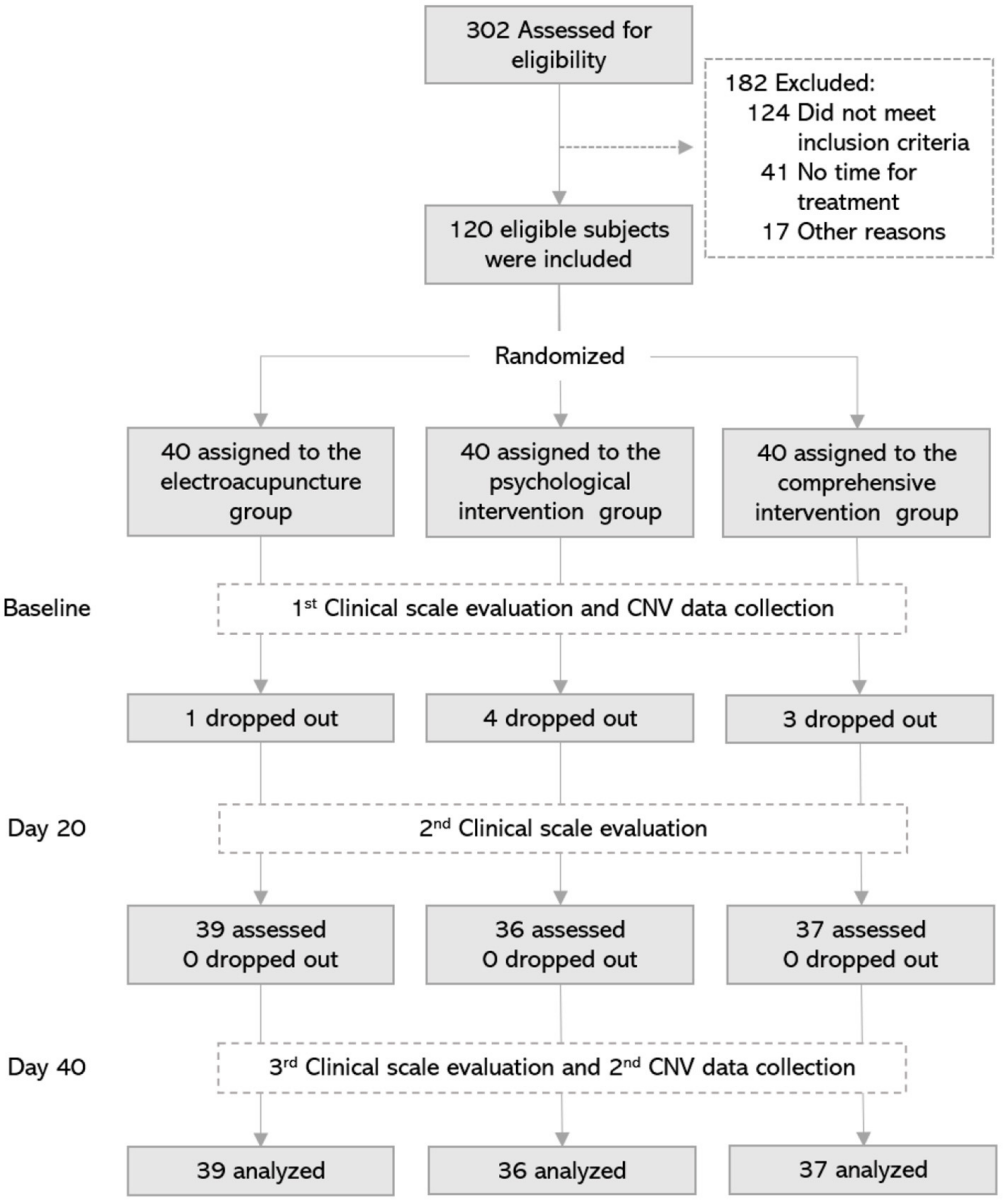
Flowchart of the screening, enrollment, randomization, assessment, and treatment. Day 20, 20th day of treatment; Day 40, 40th day of treatment; CNV, contingent negative variation.

Before the clinical trial began, all participants were informed of the purpose of the trial and signed informed consent forms. Participants were told that their demographic information would be kept confidential and that they could withdraw from the study at any trial stage. In this study, a researcher (HL) who was not involved in the experimental process used SAS 8.0 software (SAS Institute, Cary, NC, USA) to generate random number tables and group them. These random Numbers and the corresponding group information were put into an opaque envelope for sealing, and numbered on the outside of the envelope. After the patient was confirmed as enrolled, the researchers were able to open the numbered envelope to complete the grouping of the patient. These subjects were randomly assigned to one of three groups: (1) EA group; (2) PI group; (3) CI group, with 40 patients in each group (Figure 1).

All patients were blinded to group assignment. In order to better implement the blind method, we did not inform subjects of the specific content of the treatment at the time of recruitment. When they were formally enrolled in a group, they were told which treatment they would receive. Participants in each group were unaware of the other groups. All treatments were done in separate treatment rooms to avoid communication between patients. Outcome assessors and statisticians were not informed about the group allocation.

### Groups and Interventions

#### Electroacupuncture Group

**Figure 2:**
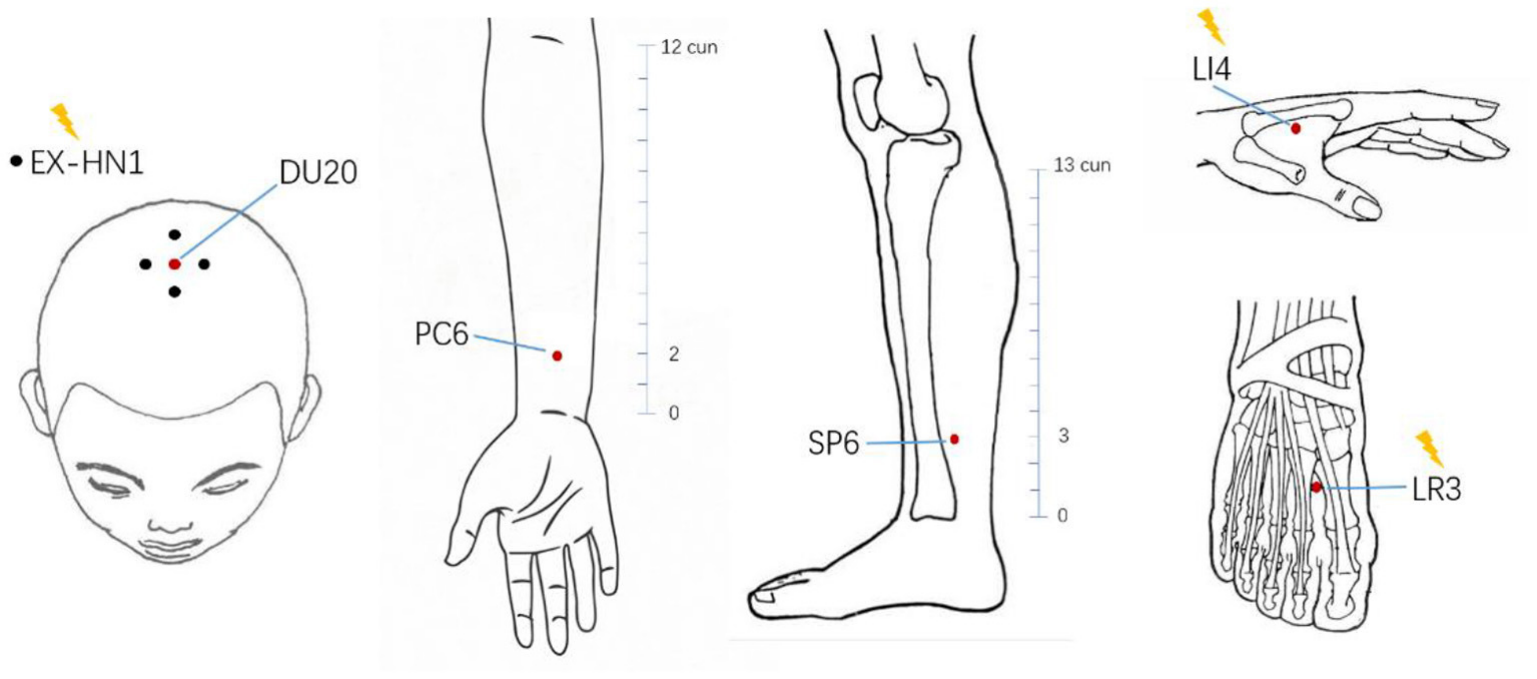
Location of acupoints in electroacupuncture group. The acupuncture points in the electroacupuncture group included six types of acupoints (a total of 13 points). The points marked by the yellow lightning bolt were the points that require electrical stimulation with electrodes attached. cun, a measure of length in traditional Chinese medicine.

Subjects in the EA group received 20 standard acupuncture treatments performed by an experienced practitioner of Traditional Chinese Medicine. Acupuncture needles were placed at Baihui (DU20), Sishencong (EX-HN1), Hegu (LI4), Taichong (LR3), Neiguan (PC6), and Sanyinjiao (SP6) acupoints according to our previous study (34). The location of acupoints was shown in Figure 2. All these acupoints were located according to the National standard of acupoints of the People's Republic of China (GB 12346-90). Huatuo brand stainless steel filiform needles (size: 0.25 × 40 mm and 0.25 × 25 mm, Suzhou Medical Supplies Factory, China) were used in the acupuncture treatment. After the needle was inserted into the skin, the needle operator performed the lifting and twisting operation to make the subject feel the sensation of “De Qi” (a sensation of the acid bilge, numbness, and pain). Then, several specific acupoints (EX-HN1, LI4, and LR3) were selected to connect with the electroacupuncture therapy instrument. Among them, two points of EX-HN1 (anterior and posterior), left LI4 and left LR3, were in one group, while the other two points of EX-HN1 (left and right), right LI4, and right LR3 were in another group. Both two groups of acupoints were electrically stimulated alternately. A total of 4 points received electrical stimulation at each time. A dilatational wave with a frequency of 10–100 Hz and wave width of 0.3 ms electrical stimulation was performed for 30 min using G6805 multi-channel electroacupuncture therapeutic apparatus (Shanghai Huayi Medical Instrument, China). The treatment time was from 3 to 4 p.m., and the treatment was once every other day, with 10 consecutive times as one course of treatment, a total of two courses.

#### Psychological Intervention Group

Subjects in the PI group were treated with cognitive-behavioral therapy (CBT) by a professional psychologist. The therapy mainly included the following four aspects: (1) The first is to understand the early experiences of Internet addicts, to investigate the root causes of their lousy personality and negative emotions. (2) Working with subjects to objectively and comprehensively analyze and evaluate the network, and then transform their cognitive concept of network dependence. (3) Working with the subject to develop a scientific and reasonable schedule to help them return to everyday life. (4) The subjects were asked to sign a contract with their family members to abstain from Internet addiction behaviors, thus gradually weakening Internet addiction behaviors. The treatment was performed every 5 days, at 4–5 p.m. for 30 min each time. Four consecutive treatments are one course of treatment, a total of two courses of treatment.

#### Comprehensive Intervention Group

Participants in the CI group received both EA and PI treatments. The frequency and method of treatment were the same as the EA group and the PI group.

### Outcome Measures

#### Clinical Assessments

Clinical assessments were conducted at baseline, the 20th day, and after completion of the last treatment. The Internet Addiction Test (IAT) developed by Kimberly Young (1) was used to assess Internet addiction severity. The Zung Self-rating Depression Scale (SDS) (35) and the 24-item Hamilton Depression Scale (HAMD) (36) were used to assess the subjects' emotional state. All the above clinical assessments were required to be conducted in a quiet environment. The subject needed to remain awake and attentive and follow the evaluator's instructions.

#### CNV Data Acquisition

CNV data were collected before treatment and on the 40th day of treatment using the MEB-9200 induction potentiometer (Nihon Kohden, Japan). Electrodes are placed following the international 10/20 EEG recording system. Considering that CNV was most often recorded at the apex of the scalp (24, 37), the recording electrode was placed at the Cz of scalp. Reference electrodes were placed on the mastoid process behind both ears, and the grounding electrode was placed at the midpoint of the forehead. The raw EEG was obtained with a sample rate of 500 Hz and the electrode impedance was <5 KΩ with skin.

The CNV-induced stimulus pattern consists of two types of signals. The first signal is S1, which is 1,000 Hz, 80 dB short sound stimulus lasting 100 ms, belonging to the prompt signal. The second signal is S2, which is the Goggle flash stimulus and belongs to the command signal. Subjects received the stimulation of these two signals alternately. After receiving S1, subjects were asked to focus their attention on anticipating the appearance of S2, and as soon as the S2 signal appeared, subjects needed to make a button response immediately to interrupt S2. The time interval between the two signals was 1.5 s, and the interval between S2 and the next S1 was 3–8 s (random). Each round was superimposed for 18 times. There were two rounds with an interval of 30 s between them. The experiment was conducted in a shielded soundproof chamber, where subjects were asked to lie flat on their beds, relax, remain awake, and focused. The whole process was examined by a regular professional doctor in the electrophysiological testing room.

#### CNV Data Processing

**Figure 3:**
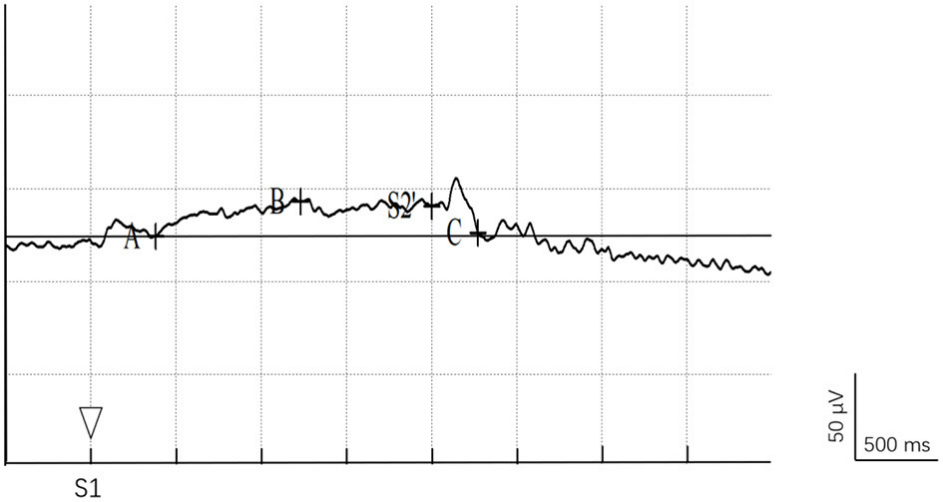
CNV waveform diagram. Point A is the point where the CNV wave leaves the baseline, point B is the peak point of the negative deflection of the E-wave, point C is the point where CNV returns to the baseline after S2.

SCAN 4.3 software was used for offline analysis to obtain the CNV for each lead and calculate the latency, amplitude, and area under the curve. All raw data were filtered (low-pass filtering 10 Hz) after removing eye movement interference and electrical artifacts. The period from 500 ms before S1 stimulus presentation to 1,000 ms after S2 stimulus presentation was superimposed, and the voltage value before 200 ms of S1 stimulus presentation was taken as the baseline. Then the CNV data were averaged and the waveform of each subject was obtained. The CNV waveform of a single subject is shown in Figure 3. The main observed indicators were the latency of point A, the average amplitude of E-wave, and the area under the curve from point A to S2 (area of A-S2') (31). CNV amplitude was measured at 500–600 ms after S1 and 50–100 ms before S2.

### Statistical Analysis

Demographic and behavioral data from each case report form was entered into a computer independently by two researchers. The data were then checked manually by another researcher. All data were locked after a blind audit and analyzed by statistical professionals with clinical trial experience. SPSS 13.0 software (IBM Corporation, NY, USA) was used for data analysis. For continuous variables, we performed a Kolmogorov–Smirnov test. Data that conformed to a normal distribution would be described in terms of mean and standard deviation. For categorical variables, they were expressed in terms of frequency or percentage. To compare the differences between the three groups of data, a one-way analysis of variance (ANOVA) was used for comparison. If ANOVA results showed significant differences between the three groups, pairwise comparisons were performed using the Least Significant Difference (LSD) test. For the same group of changes at baseline and post-treatment, the paired *t-*test was used. The chi-squared test was used for comparison of dichotomous data and the Ridit test for ranked data. Pearson correlation analysis was also used to analyze the correlation between electrophysiological data and clinical assessment data. A *p* < 0.05 was considered statistically significant.

## Results

### Demographic Characteristics

A total of 120 IAD subjects were enrolled and randomized into three groups. However, in the follow-up study, 1 case fell off in the electroacupuncture group, 4 cases fell off in the psychological group, and 3 cases fell off in the comprehensive group. Data from 112 cases were included in the statistical analysis. Of the 112 IAD subjects included in the data analysis, 73 were males and 39 females. As shown in Table 1, there was no significant difference in male/female distribution, age, Internet age, or surfing time among the groups at baseline (*P* > 0.05).

**Table 1:** Demographic characteristics of the participants with IAD (Mean ± SD).

	**EA group (*n* = 39)**	**PI group (*n* = 36)**	**CI group (*n* = 37)**	***P*-value**
Age (years)	21.00 ± 1.97	22.53 ± 2.26	22.41 ± 2.10	>0.05
Gender (male/female)	24/15	24/12	25/12	>0.05
Internet age (years)	4.73 ± 1.91	4.19 ± 2.01	4.76 ± 2.10	>0.05
Surfing time (hours/day)	6.13 ± 1.97	6.28 ± 2.47	6.22 ± 1.84	>0.05

*EA, electropuncture; PI, psychological intervention; CI, comprehensive intervention*.

### Internet Addiction Self-Rating Scale

**Figure 4:**
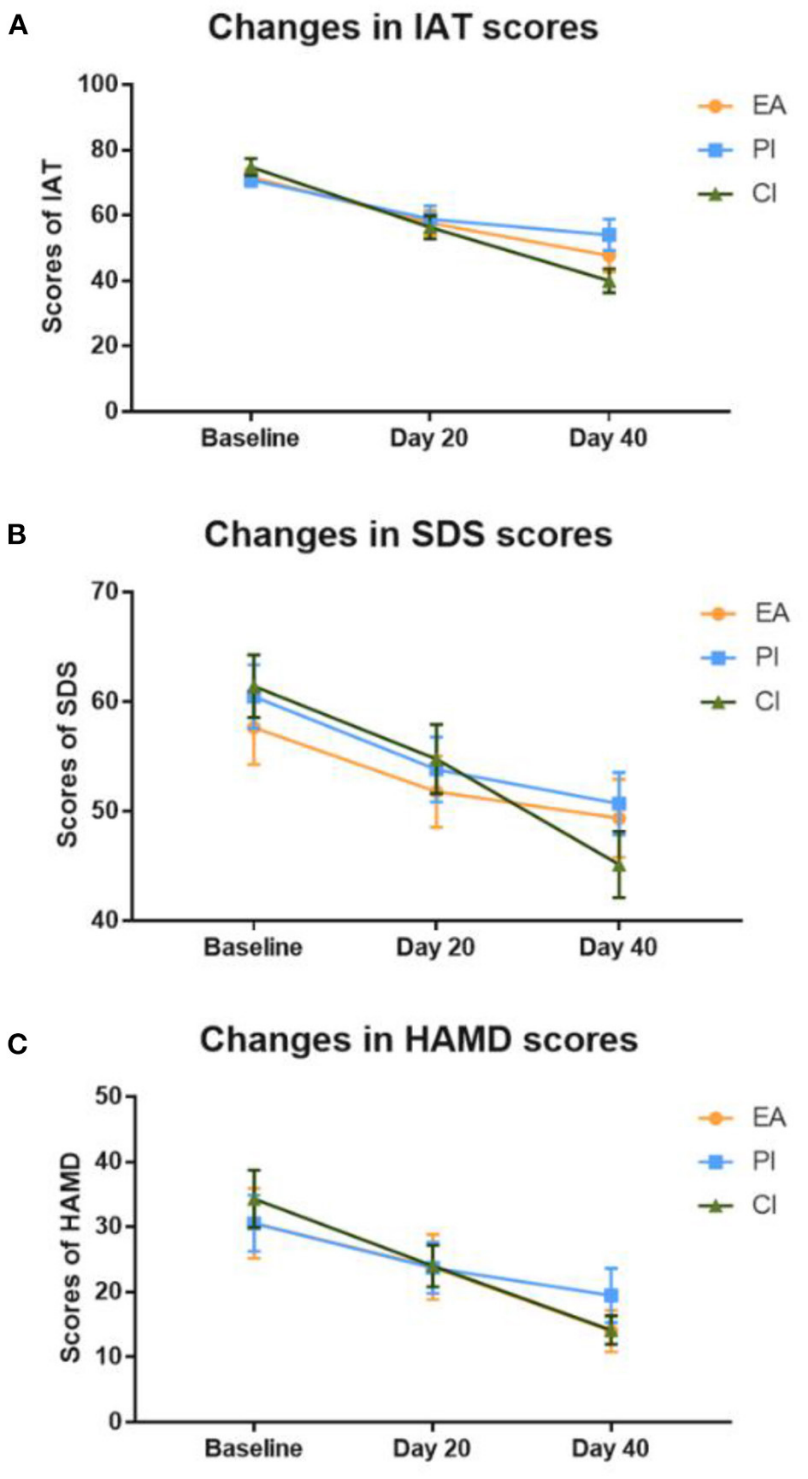
Changes in the score of the clinical evaluation scale. **(A)** Changes of IAT scores in the three groups at different treatment time points. **(B)** Changes of SDS scores in the three groups at different treatment time points. **(C)** Changes of HAMD scores in the three groups at different treatment time points. EA, electropuncture; PI, psychological intervention; CI, comprehensive intervention; IAT, Young's Internet addiction test; SDS, Zung Self-rating depression scale; HAMD, Hamilton Depression Scale; Day 20, 20th day of treatment; Day 40, 40th day of treatment.

The primary outcome for the severity of Internet addiction assessment was IAT. As shown in Figure 4 and Table 2, there was no significant difference in the scores of IAT among the groups at baseline (*P* > 0.05). With the increase of treatment time, the IAT scores of the three groups showed a significant decreasing trend, among which the IAT scores after 40 days of treatment were significantly lower than those at baseline and 20 days of treatment (*P* < 0.05). After 40 days of treatment, IAT scores in the comprehensive group were significantly lower than those in the other two groups (*P* < 0.05), while IAT scores in the electroacupuncture group were lower than those in the psychological group (*P* < 0.05).

**Table 2:** Comparison of clinical scale scores of IAD subjects in three groups (Mean ± SD).

	**EA group (*n* = 39)**	**PI group (*n* = 36)**	**CI group (*n* = 37)**	**Total *P*-value**	**EA vs. PI *P*-value**	**CI vs. PI *P*-value**	**EA vs. CI *P*-value**
**IAT**							
Baseline	71.67 ± 7.62	70.89 ± 6.04	74.81 ± 7.63	>0.05	—	—	—
Day 20	57.74 ± 12.08^a^	58.89 ± 11.93^a^	56.35 ± 10.59^a^	>0.05	—	—	—
Day 40	47.72 ± 15.46^a^^b^	54.08 ± 14.23^a^^b^	40.05 ± 10.92^a^^b^	**<0.001**	**0.047**	**<0.001**	**0.016**
**SDS**							
Baseline	57.64 ± 10.42	60.47 ± 8.58	61.41 ± 8.52	>0.05	—	—	—
Day 20	51.79 ± 10.03^a^	53.81 ± 8.75^a^	54.76 ± 9.42^a^	>0.05	—	—	—
Day 40	49.36 ± 10.99^a^	50.69 ± 8.43^a^^b^	45.14 ± 9.08^a^^b^	**0.038**	>0/05	**0.015**	>0.05
**HAMD**							
Baseline	30.56 ± 16.57	30.58 ± 12.78	34.32 ± 13.18	>0.05	—	—	—
Day 20	23.85 ± 15.45^a^	23.72 ± 11.55^a^	24.00 ± 9.59^a^	>0.05	—	—	—
Day 40	14.00 ± 9.83^a^^b^	19.47 ± 12.29^a^^b^	14.16 ± 6.60^a^^b^	**0.028**	**0.018**	**0.023**	>0.05

a *Compared with the baseline, P < 0.05*.

b *Compared with the Day 20, P < 0.05*.

*IAT, Young's Internet addiction test; SDS, Zung self-rating depression scale; HAMD, Hamilton depression scale; Day 20, 20th day of treatment; Day 40, 40th day of treatment. Statistically significant data were shown in bold*.

### Emotional State

SDS and HAMD scores can reflect the emotional state of IAD individuals. As shown in Figure 4 and Table 2, there was no significant difference in SDS and HAMD scores among the groups at baseline (*P* > 0.05). SDS and HAMD scores in each group showed a downward trend with the increase of treatment time. After 20 days of treatment, both SDS and HAMD scores decreased significantly in all three groups (*P* < 0.05). On the 40th day of treatment, each group's HAMD scores were significantly lower than those on the 20th day of treatment (*P* < 0.05). After comparison among the three groups, the HAMD score of the comprehensive group and the electroacupuncture group after 40 days of treatment was significantly lower than that of the psychological group (*P* < 0.05). In terms of SDS scores, only the comprehensive group and the psychological group scored significantly lower at day 40 than at day 20 of treatment (*P* < 0.05). After the intergroup analysis, the comprehensive group's SDS score was significantly lower than that of the psychological group on the 40th day of treatment (*P* < 0.05).

### CNV Data

#### Latency of Point A

There was no significant difference in the latency of CNV before treatment in each group (*P* > 0.05). After 40 days of treatment, there was still no significant difference in the latency of CNV among these groups (*P* > 0.05). Compared with each group before treatment, there was no significant change in the CNV incubation period 40 days after treatment (*P* > 0.05; see Table 3).

**Table 3:** Comparison of CNV components in three groups (Mean ± SD).

	**EA group (*n* = 39)**	**PI group (*n* = 36)**	**CI group (*n* = 37)**	***P*-value**
**Latency (ms)**				
Baseline	368.46 ± 113.86	385.69 ± 115.70	376.08 ± 89.19	0.573
Day 40	365.85 ± 111.66	406.81 ± 89.39	371.22 ± 105.30	0.164
**Amplitude (μV)**				
Baseline	12.46 ± 8.86	9.69 ± 12.09	9.70 ± 6.05	0.329
Day 40	17.13 ± 9.10^a^	15.89 ± 15.04^a^	17.59 ± 15.21^a^	0.853
**Area of A-S2' (μV·ms)**				
Baseline	19.10 ± 13.36	12.58 ± 12.60	14.33 ± 10.64	0.190
Day 40	21.92 ± 17.05	20.28 ± 21.44^a^	21.30 ± 20.79	0.937

a *Compared with baseline, P < 0.05*.

*EA, electropuncture; PI, psychological intervention; CI, comprehensive intervention*.

#### Amplitude

There was no significant difference in CNV amplitude between the groups at baseline (*P* > 0.05). On the 40th day of treatment, the amplitude of CNV in each group was significantly higher than that before treatment (*P* < 0.05). However, the CNV amplitude of the three groups was compared between groups after 40 days of treatment, and the difference was not statistically significant (*P* > 0.05; see Table 3).

#### Area of A-S2'

There was no significant difference in the CNV A-S2' wave area before treatment (*P* > 0.05). On the 40th day of treatment, only the psychological group showed a significant increase in the CNV A-S2' wave area compared with that before treatment (*P* < 0.05). In contrast, the other two groups showed no significant change (P>0.05). On the 40th day of treatment, there was no significant difference in the CNV A-S2' wave area among the three groups (*P* > 0.05; see Table 3).

### Correlation Between CNV and Clinical Scale Scores

Table 4 lists the correlations between the main indicators of CNV and clinical scale scores at baseline. As shown in the table, the amplitude and area of CNV are significantly positively correlated (*r* = 0.699, *P* < 0.01). IAT scores were positively correlated with SDS scores (*r* = 0.205, *P* < 0.05) and HAMD scores (*r* = 0.269, *P* < 0.01) respectively, but negatively correlated with CNV latency (*r* = −0.198, *P* < 0.05). Table 5 shows the correlation between changes in CNV and changes in clinical scores in each group after treatment. Only the variation of amplitude in the CI group was negatively correlated with the change of the HAMD score (*r* = −0.626, *P* < 0.01).

**Table 4:** Pearson correlations between CNV and clinical measures at baseline.

	**Latency**	**Amplitude**	**Area of A-S2'**	**IAT**	**SDS**	**HAMD**
Latency	1	−0.069	−0.089	–0.198^*^	−0.177	−0.108
Amplitude	−0.069	1	0.699^**^	0.047	0.029	0.067
Area of A-S2'	−0.089	0.699^**^	1	0.068	0.027	0.120
IAT	–0.198^*^	0.047	0.068	1	0.205^*^	0.269^**^
SDS	−0.177	0.029	0.027	0.205^*^	1	0.428^**^
HAMD	−0.108	0.067	0.120	0.269^**^	0.428^**^	1

* *P < 0.05;*

** *P < 0.01; IAT, young's internet addiction test; SDS, zung self-rating depression scale; HAMD, Hamilton depression scale. Statistically significant data were shown in bold*.

**Table 5:** Pearson correlations between CNV change and clinical index change.

	**Change in latency**	**Change in amplitude**	**Change in A-S2' area**
**EA**			
Change in IAT	0.009	–0.174	–0.016
Change in SDS	–0.206	0.251	0.236
Change in HAMD	–0.147	–0.111	0.013
**PI**			
Change in IAT	–0.044	–0.009	–0.107
Change in SDS	0.016	0.016	–0.099
Change in HAMD	–0.021	0.124	–0.044
**CI**			
Change in IAT	–0.290	–0.054	0.182
Change in SDS	0.014	0.307	0.278
Change in HAMD	–0.022	−0.626^**^	−0.103

** *P < 0.01*.

*EA, electropuncture; PI, psychological intervention; CI, comprehensive intervention; IAT, Young's Internet addiction test; SDS, Zung Self-rating depression scale; HAMD, Hamilton depression scale. Statistically significant data were shown in bold*.

## Discussion

In the present study, we observed the differences in the regulatory effects of EA and PI on the severity of Internet addiction and depression in IAD patients and the changes in CNV composition in IAD patients before and after treatment. Our results showed that both EA and PI could effectively reduce the score of Internet addiction and depression scale in IAD patients, and CI showed better efficacy than the other groups. Besides, our ERP results suggested that the increased CNV amplitude was associated with an improvement in depression. These findings confirmed CI's superiority in the treatment of IAD and might provide new ideas for studying the IAD intervention mechanism.

Young's IAT is the most commonly used questionnaire for assessing Internet addiction (38, 39). There are 20 items on this scale, which adopts the five-level scoring method. The higher the total score is, the more serious the degree of Internet addiction is. According to the IAT score, the tested subjects can be classified into normal (0–30), mild (31–49), moderate (50–79), and severe (80–100) types (38). In our study, the subjects included were moderate to severe IAD. With the increase of treatment time, the three treatment measures' IAT total scores showed a continuous downward trend. This result is consistent with our previous findings (15). After 40 days of treatment, EA improved the severity of IAD more than psychological intervention, and EA combined with PI showed better results than monotherapy. The results showed that the CI had a synergistic effect. Although there are few reports on acupuncture combined with psychological therapy in IAD at present, the synergistic effect of acupuncture and psychological intervention has been proved in studies on post-stroke depression (40), post-stroke speech disorder (41), and chronic fatigue syndrome (42). Acupuncture is an exogenous stimulus, often accompanied by pain. Most people, especially young people, are afraid of pain. Psychological interventions can help ease their fear of acupuncture and improve the effectiveness of treatment.

In this study, two scales were selected to assess depression status. Both SDS and HAMD are widely used in clinical and scientific research (43, 44). The correlation analysis results showed that IAT scores of IAD patients were positively correlated with depression scales scores. This result was consistent with the results of others (18, 19). After 20 days of treatment, SDS and HAMD scores in all three groups were significantly reduced, consistent with our expected results. After 40 days of treatment, SDS and HAMD scores in all three groups were significantly lower than those in the baseline period, consistent with our expected results. Interestingly, the intergroup analysis of SDS showed that after 40 days of treatment, only CI was superior to PI, while HAMD results showed that both EA and CI were superior to PI. The reason may be related to the difference of questionnaire items and the subjective intention of the person who filled out the form at that time. We cannot guarantee that the person filling in the form can fill in the form faithfully every time for a subjective self-rating scale. Researchers have pointed out that these as the gold standard of measurement tools, psychological measurement, and concept have some minor flaws (45, 46). That could be another reason for the difference in our results. Although we cannot determine the difference in efficacy between EA and CI in improving depressive symptoms, we found that improvement in depressive symptoms in IAD patients was associated with improvement in Internet addiction severity, suggesting that improving the depressive symptoms was indeed an approach for treating IAD.

CNV is a kind of evoked brain potential closely related to psychiatry and psychology (47). It is produced under two specific stimulus conditions (S1 and S2), reflecting the connection between the two stimuli. Because the CNV amplitude in the scalp record at the highest of the central band, so the CNV waveform at Cz was used as analysis indicators. Previous studies have found that the CNV amplitude decreases in patients with depression (48, 49) and IGD (32). In our study, we found that the CNV amplitude increased in all three groups of IAD patients after treatment, accompanied by a decrease in depression scale scores. Most importantly, Pearson correlation analysis of the CI group indicated that the variation of the HAMD score was significantly negatively correlated with the amplitude. This finding provided evidence for the role of CI in regulating the depressive state in IAD patients.

Another important indicator of CNV is latency. For this CNV feature, most CNV studies focus on the peak latency. In our study, we chose the latency of point A as the main observation feature of the early CNV. In fact, both types of latency can reflect the level of sustained attention to the stimulus (50, 51). We found that among the three CNV indicators at baseline, only the latency of point A was negatively correlated with the IAT score. In contrast, the other two indexes in our study were not correlated with IAT, SDS, and HAMD scores. Unfortunately, our study did not observe differences in the latency of CNV between the three groups after treatment. In addition, our previous study found that there was no difference in the latency of point A between IAD subjects and healthy subjects. Based on the results of the two studies, we believed that the incubation period of point A might not be an electrophysiological feature of IAD. As there are few CNV studies on IAD at present, we have not found other studies that are consistent with our research results. In the CNV study of depression, most researchers have observed that the CNV latency of depressed patients is longer than normal people (52). However, another research found no statistically significant difference between the CNV incubation period of depressed patients and that of normal people (53). These inconsistent findings suggest that the latency of CNV might not be a specific feature of subjects with IAD with depressive status. We also found an interesting result that the area of A-S2' in the PI group increased after treatment, while there was no significant change in the EA and CI groups. However, among the clinical scale assessment results, PI had the weakest effect on improving Internet addiction severity and depression in IAD patients in the three groups. Some studies have found that the wave area of CNV is related to cognitive function. In the population with impaired cognitive function, the wave area of CNV is significantly lower than that of healthy people (54). The CBT used in the PI group is a recognized therapy that can improve patients' cognitive function, so the increase of CNV wave area in the PI group might be related to the improvement of patients' cognitive function. Most scholars believed that the cognitive function of IAD was impaired (55, 56). In our study, although the CI group also received CBT treatment, their CNV wave area did not change significantly, which might be related to the treatment regimen that the CI group received. The combined use of the two therapies might weaken CBT's improvement on cognitive function, so there was no significant change in the CNV wave area of the EA group and the CI group.

To the best of our knowledge, the present study is the first to examine the CNV changes in IAD patients with EA combined with CBT. As expected, CI was more effective than EA and PI in improving the severity of Internet addiction and depressive symptoms among the three treatment measures. At the same time, EA was more effective than PI in improving the severity of Internet addiction. However, the efficacy of EA and PI in improving depressive symptoms remains to be further verified. Moreover, the latency of CNV was negatively correlated with IAT scores, indicating that the CNV latency might be an important feature of IAD's electrophysiology. Besides, the increase of CNV amplitude also reflected the improvement of IAD depressive state by acupuncture or CBT.

### Limitations

Several limitations of this study deserve to be considered. First, CNV was measured on only one electrode, not on the extensive scalp. Because most studies believed that CNV amplitude was most obvious when measured on Cz, we chose one electrode for preliminary exploration. We conducted CNV research on other electrodes in the future according to the results of this study. Secondly, we did not set a blank control group, which could not rule out IAD's self-healing nature. Since the purpose of this study was to screen out the best treatment that could improve the severity of Internet addiction and depressive symptoms, we did not set up such a blank control group. Thirdly, our study did not limit the degree of depressive symptoms of IAD subjects in the recruitment stage. This means that different IAD subjects had different levels of depression. In the next stage, we will conduct an in-depth study on IAD population with different levels of depression according to the results of this study.

## Conclusion

CI therapy can effectively improve the severity of Internet addiction and depressive symptoms in IAD patients, which is better than EA and PI alone. Simultaneously, the increase in CNV amplitude might be the underlying neurophysiological mechanism by which CI improves depression and cognitive function in IAD patients.

## Data Availability Statement

The raw data supporting the conclusions of this article will be made available by the authors, without undue reservation.

## Ethics Statement

The studies involving human participants were reviewed and approved by the Sichuan Traditional Chinese Medicine Regional Ethics Review Committee in China. The patients/participants provided their written informed consent to participate in this study.

## Author Contributions

TZ and HL designed the study and acquired the funding in China. JW, QH, MQ, YC, and YT were involved in the recruitment and treatment of patients. WP conducted the statistical analysis. WP wrote the first draft of the manuscript. TZ and YW revised the manuscript. All authors have approved the final manuscript.

## Funding

This work was supported by the National Natural Science Foundation of China (81072852 and 81574047), the Key R&D Project of Sichuan Province (2019YFS0175), the Xinglin Scholars Scientific Research Promotion Program of Chengdu University of Traditional Chinese Medicine (XSGG2019007), and the Training Funds of Academic and Technical Leader in Sichuan Province.

## Conflict of Interest

The authors declare that the research was conducted in the absence of any commercial or financial relationships that could be construed as a potential conflict of interest.

## Publisher's Note

All claims expressed in this article are solely those of the authors and do not necessarily represent those of their affiliated organizations, or those of the publisher, the editors and the reviewers. Any product that may be evaluated in this article, or claim that may be made by its manufacturer, is not guaranteed or endorsed by the publisher.
